# Crystal structure of 1,3,5-trimethyl-2,4-di­nitro­benzene

**DOI:** 10.1107/S2056989015014243

**Published:** 2015-08-22

**Authors:** Ouarda Brihi, Noudjoud Hamdouni, Ali Boudjada, Jean Meinnel

**Affiliations:** a Laboratoire de Cristallographie, Département de Physique, Université Mentouri-Constantine, 25000 Constantine, Algeria; b UMR 6226 CNRS–Université Rennes 1 ‘Sciences Chimiques de Rennes’, Equipe ‘Matière Condensée et Systèmes Electroactifs’, 263 Avenue du Général Leclerc, F-35042 Rennes, France

**Keywords:** crystal structure, di­nitro­benzene, weak C—H⋯O inter­action

## Abstract

In the title compound, C_9_H_10_N_2_O_4_, the planes of the nitro groups subtend dihedral angles of 55.04 (15) and 63.23 (15)° with that of the aromatic ring. These tilts are in opposite senses and the mol­ecule possesses approximate mirror symmetry about a plane normal to the mol­ecule. In the crystal, mol­ecules form stacks in the [100] direction with adjacent mol­ecules related by translation, although the centroid–centroid separation of 4.136 (5) Å is probably too long to regard as a significant aromatic π–π stacking inter­action. An extremely weak C—H⋯O inter­action is also present.

## Related literature   

For the structures and properties of related compounds, see: Tazi *et al.* (1995 ▸); Hernandez *et al.* (2003 ▸). 
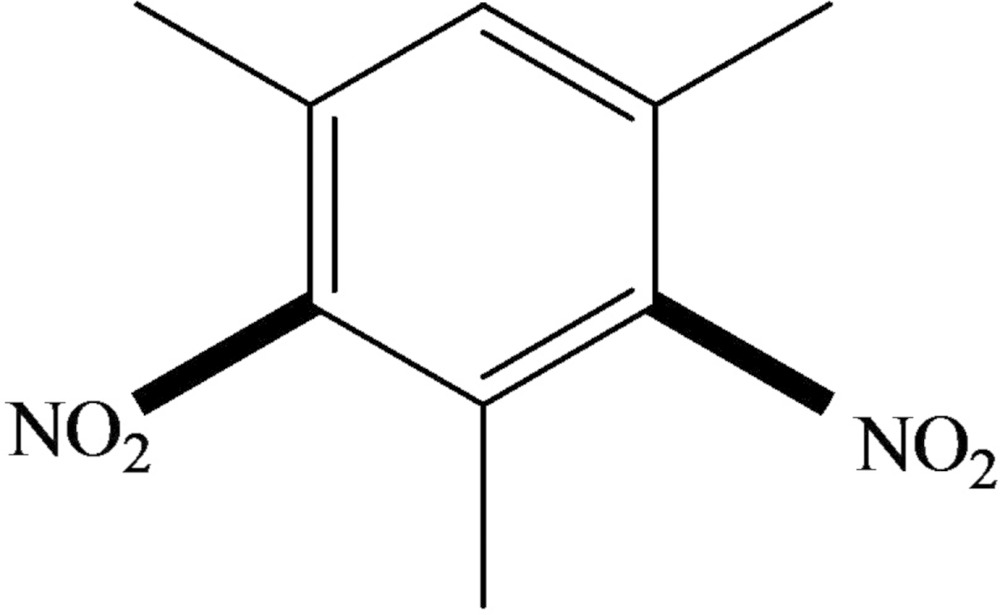


## Experimental   

### Crystal data  

C_9_H_10_N_2_O_4_
*M* *_r_* = 210.19
Orthorhombic, 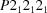

*a* = 4.136 (5) Å
*b* = 13.916 (5) Å
*c* = 17.194 (5) Å
*V* = 989.6 (13) Å^3^
*Z* = 4
Mo *K*α radiation
μ = 0.11 mm^−1^
*T* = 293 K
0.1 × 0.08 × 0.08 mm

### Data collection  

Oxford Diffraction Xcalibur diffractometer
Absorption correction: multi-scan (*CrysAlis PRO*; Oxford Diffraction, 2010 ▸) *T*_min_ = 0.618, *T*_max_ = 1.000
3941 measured reflections
2730 independent reflections
1302 reflections with *I* > 2σ(*I*)
*R*_int_ = 0.032
Standard reflections: ?

### Refinement  

*R*[*F* ^2^ > 2σ(*F* ^2^)] = 0.053
*wR*(*F* ^2^) = 0.113
*S* = 0.93
2730 reflections
139 parameters
H atoms treated by a mixture of independent and constrained refinement
Δρ_max_ = 0.12 e Å^−3^
Δρ_min_ = −0.15 e Å^−3^

### 

Data collection: *CrysAlis RED* (Oxford Diffraction, 2002 ▸); cell refinement: *CrysAlis RED*; data reduction: *CrysAlis RED*; program(s) used to solve structure: *SIR2002* (Burla *et al.*, 2005 ▸); program(s) used to refine structure: *SHELXL97* (Sheldrick, 2008 ▸); molecular graphics: *CAMERON* (Watkin *et al.*, 1996 ▸); software used to prepare material for publication: *WinGX* (Farrugia, 2012 ▸).

## Supplementary Material

Crystal structure: contains datablock(s) ouarda, I. DOI: 10.1107/S2056989015014243/hb7463sup1.cif

Structure factors: contains datablock(s) I. DOI: 10.1107/S2056989015014243/hb7463Isup2.hkl

Supporting information file. DOI: 10.1107/S2056989015014243/hb7463Isup3.cml

**Fig. 1.**
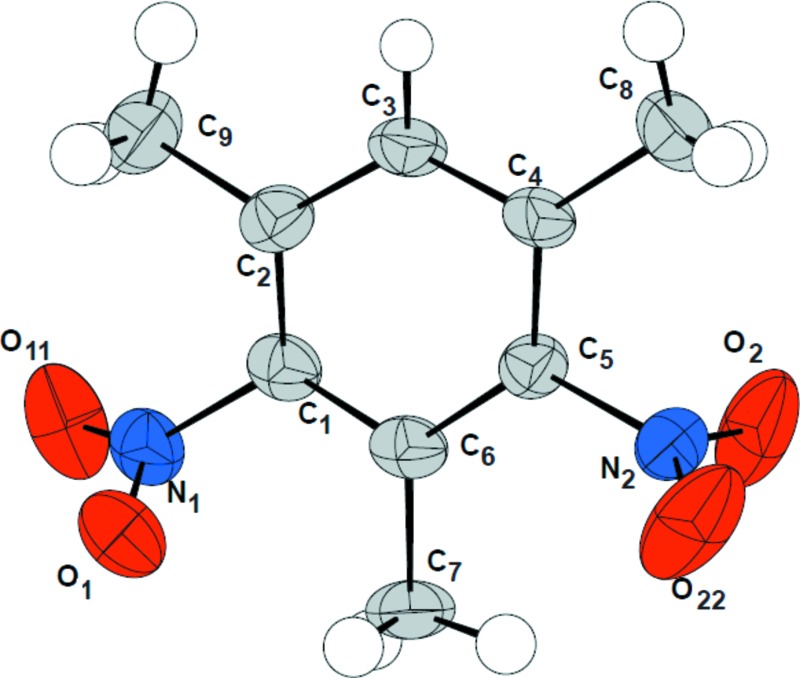
The molecular structure of (I) with displacement ellipsoids drawn at the 50% probability level.

**Fig. 2.**
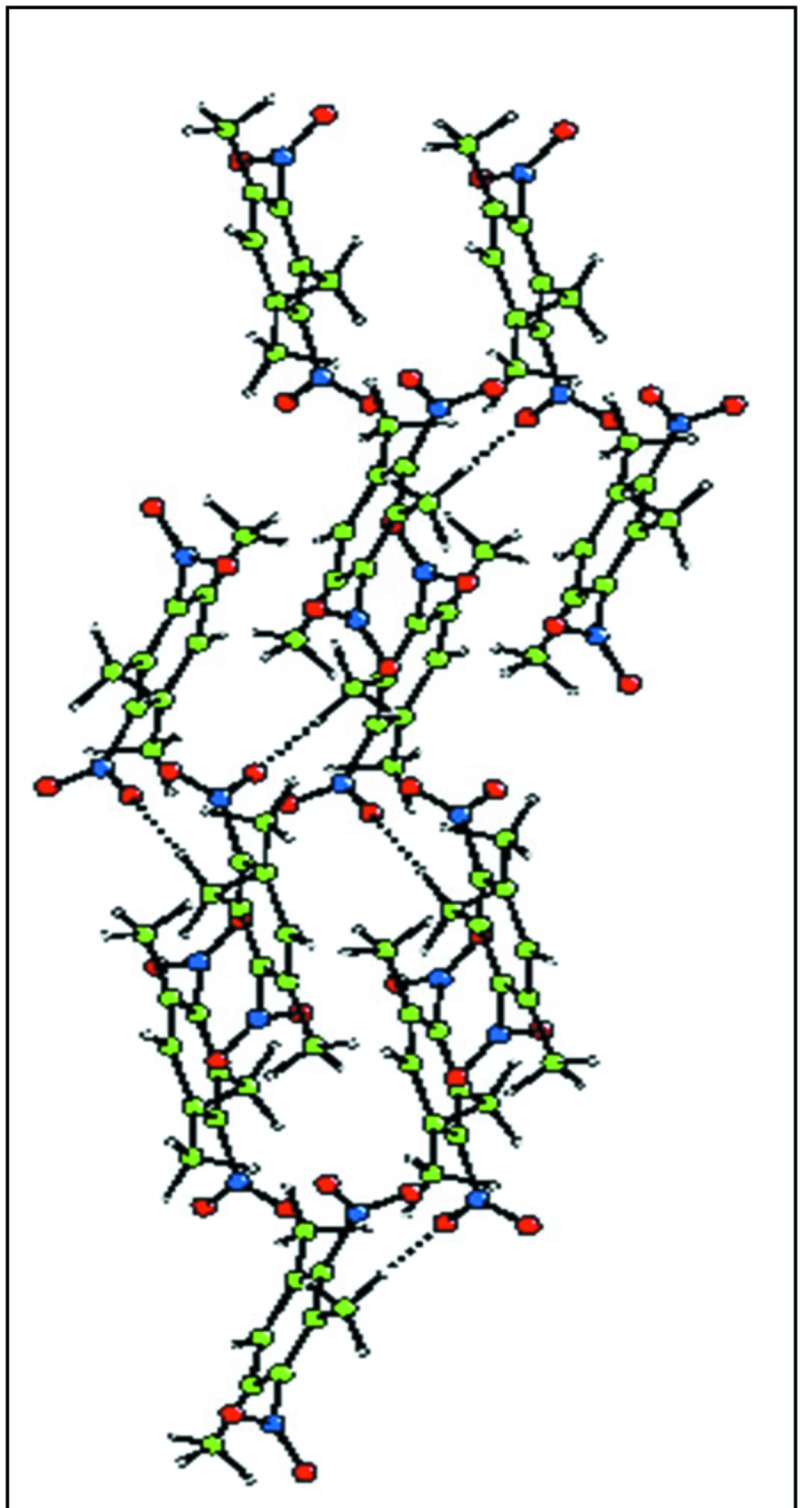
The crystal packing of (I) at 293 K, along the b axis.

. DOI: 10.1107/S2056989015014243/hb7463fig1.tif
The mol­ecular structure of (I) with displacement ellipsoids drawn at the 50% probability level.

b . DOI: 10.1107/S2056989015014243/hb7463fig2.tif
The crystal packing of (I) at 293 K, along the *b* axis.

**Fig. 3.**
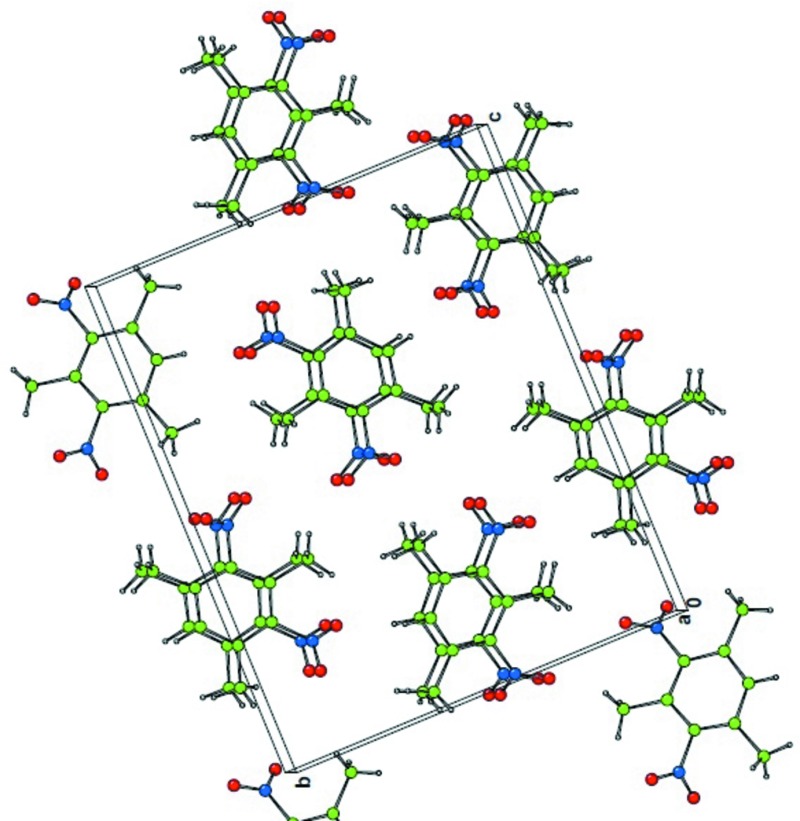
The crystal packing of (I) at 293 K, according to the direction [100].

. DOI: 10.1107/S2056989015014243/hb7463fig3.tif
The crystal packing of (I) at 293 K, according to the direction [100].

CCDC reference: 1415489

Additional supporting information:  crystallographic information; 3D view; checkCIF report

## Figures and Tables

**Table 1:** Hydrogen-bond geometry (, )

*D*H*A*	*D*H	H*A*	*D* *A*	*D*H*A*
C7H7*C*O1^i^	0.96	2.60	3.232(4)	124

Symmetry code: (i) 
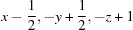
.

## supplementary crystallographic information

### S1. Experimental

The commercially available compound (Sigma-Aldrich) Was recrystallized from ethanol solution.

### S2. Refinement

All non-H atoms were refined with anisotropic atomic displacement parameters. All H atoms were localized in a Fourier maps but introduced in calculated positions and treated as riding on their parent C atoms with C_aryl_—H_aryl_=0.93 Å; C_methyl_—H_methyl_=0.96 Å and *U*_iso_(H_methyl_)=1.5*U*_eq_(C_methyl_) or *U*_iso_(H_aryl_)=1.2*U*_eq_(C_aryl_). The atoms of benzene cycle present parameters of atomic displacements weaker than those of the substituent atoms.

### Crystal data

**Table:**

C_9_H_10_N_2_O_4_	*F*(000) = 440
*M**_r_* = 210.19	*D*_x_ = 1.411 Mg m^−^^3^
Orthorhombic, *P*2_1_2_1_2_1_	Mo *K*α radiation, λ = 0.71073 Å
Hall symbol: P 2ac 2ab	Cell parameters from 1062 reflections
*a* = 4.136 (5) Å	θ = 3.8–25.0°
*b* = 13.916 (5) Å	µ = 0.11 mm^−^^1^
*c* = 17.194 (5) Å	*T* = 293 K
*V* = 989.6 (13) Å^3^	Needle, colourless
*Z* = 4	0.1 × 0.08 × 0.08 mm

### Data collection

**Table:**

Oxford Diffraction Xcalibur diffractometer	2730 independent reflections
Radiation source: Enhance (Mo) X-ray Source	1302 reflections with *I* > 2σ(*I*)
Graphite monochromator	*R*_int_ = 0.032
CCD scans	θ_max_ = 32.2°, θ_min_ = 3.2°
Absorption correction: multi-scan (*CrysAlis PRO*; Oxford Diffraction, 2010)	*h* = −5→3
*T*_min_ = 0.618, *T*_max_ = 1.000	*k* = −19→17
3941 measured reflections	*l* = −25→16

### Refinement

**Table:**

Refinement on *F*^2^	Primary atom site location: structure-invariant direct methods
Least-squares matrix: full	Secondary atom site location: difference Fourier map
*R*[*F*^2^ > 2σ(*F*^2^)] = 0.053	Hydrogen site location: inferred from neighbouring sites
*wR*(*F*^2^) = 0.113	H atoms treated by a mixture of independent and constrained refinement
*S* = 0.93	*w* = 1/[σ^2^(*F*_o_^2^) + (0.0312*P*)^2^] where *P* = (*F*_o_^2^ + 2*F*_c_^2^)/3
2730 reflections	(Δ/σ)_max_ < 0.001
139 parameters	Δρ_max_ = 0.12 e Å^−^^3^
0 restraints	Δρ_min_ = −0.15 e Å^−^^3^

### Special details

**Table:**

Experimental. Absorption correction: CrysAlisPro, Empirical absorption correction using spherical harmonics, implemented in SCALE3 ABSPACK scaling algorithm.
Geometry. Bond distances, angles *etc*. have been calculated using the rounded fractional coordinates. All su's are estimated from the variances of the (full) variance-covariance matrix. The cell e.s.d.'s are taken into account in the estimation of distances, angles and torsion angles
Refinement. Refinement of *F*^2^ against ALL reflections. The weighted *R*-factor *wR* and goodness of fit *S* are based on *F*^2^, conventional *R*-factors *R* are based on *F*, with *F* set to zero for negative *F*^2^. The threshold expression of *F*^2^ > σ(*F*^2^) is used only for calculating *R*-factors(gt) *etc*. and is not relevant to the choice of reflections for refinement. *R*-factors based on *F*^2^ are statistically about twice as large as those based on *F*, and *R*- factors based on ALL data will be even larger.

### Fractional atomic coordinates and isotropic or equivalent isotropic displacement parameters (Å^2^)

**Table:**

	*x*	*y*	*z*	*U*_iso_*/*U*_eq_	
O1	0.5095 (6)	0.13682 (16)	0.51948 (12)	0.0830 (9)	
O2	0.4780 (6)	0.14379 (17)	0.16111 (14)	0.0975 (10)	
O11	0.1589 (6)	0.0227 (2)	0.51482 (12)	0.0930 (9)	
O22	0.8082 (7)	0.22717 (18)	0.22671 (15)	0.1057 (11)	
N1	0.3785 (6)	0.06911 (19)	0.48712 (13)	0.0612 (9)	
N2	0.6374 (6)	0.15688 (17)	0.21810 (14)	0.0576 (8)	
C1	0.4961 (5)	0.04214 (19)	0.40898 (13)	0.0446 (8)	
C2	0.5978 (6)	−0.05263 (18)	0.39734 (15)	0.0482 (8)	
C3	0.7215 (6)	−0.07338 (17)	0.32487 (16)	0.0501 (9)	
C4	0.7380 (6)	−0.00732 (19)	0.26478 (14)	0.0466 (8)	
C5	0.6263 (6)	0.08533 (17)	0.28156 (15)	0.0439 (8)	
C6	0.5084 (5)	0.11344 (17)	0.35299 (14)	0.0435 (8)	
C7	0.3848 (8)	0.21448 (18)	0.36780 (16)	0.0664 (11)	
C8	0.8759 (7)	−0.0350 (2)	0.18697 (15)	0.0632 (10)	
C9	0.5834 (7)	−0.1295 (2)	0.45897 (17)	0.0701 (11)	
H3	0.79817	−0.13520	0.31580	0.0601*	
H7A	0.30028	0.24079	0.32036	0.0995*	
H7B	0.55903	0.25404	0.38611	0.0995*	
H7C	0.21682	0.21253	0.40631	0.0995*	
H8A	1.00516	0.01682	0.16721	0.0947*	
H8B	0.70269	−0.04818	0.15135	0.0947*	
H8C	1.00773	−0.09134	0.19269	0.0947*	
H9A	0.66925	−0.10482	0.50682	0.1050*	
H9B	0.70885	−0.18397	0.44265	0.1050*	
H9C	0.36286	−0.14884	0.46668	0.1050*	

### Atomic displacement parameters (Å^2^)

**Table:**

	*U*^11^	*U*^22^	*U*^33^	*U*^12^	*U*^13^	*U*^23^
O1	0.1139 (17)	0.0722 (16)	0.0629 (13)	−0.0037 (15)	−0.0125 (13)	−0.0276 (12)
O2	0.130 (2)	0.0873 (18)	0.0751 (14)	−0.0364 (15)	−0.0386 (16)	0.0295 (14)
O11	0.0756 (14)	0.129 (2)	0.0743 (14)	−0.0118 (16)	0.0166 (12)	−0.0084 (15)
O22	0.126 (2)	0.0670 (16)	0.124 (2)	−0.0504 (15)	−0.0339 (17)	0.0323 (15)
N1	0.0600 (14)	0.0690 (18)	0.0547 (14)	0.0088 (14)	−0.0066 (13)	−0.0055 (14)
N2	0.0685 (15)	0.0394 (13)	0.0648 (15)	−0.0027 (12)	−0.0067 (14)	0.0032 (13)
C1	0.0449 (13)	0.0466 (14)	0.0424 (13)	−0.0009 (12)	−0.0070 (12)	−0.0089 (12)
C2	0.0508 (14)	0.0394 (14)	0.0545 (15)	−0.0036 (12)	−0.0098 (13)	−0.0029 (13)
C3	0.0570 (16)	0.0294 (12)	0.0639 (17)	0.0000 (11)	−0.0025 (14)	−0.0069 (13)
C4	0.0507 (14)	0.0362 (14)	0.0529 (14)	−0.0032 (11)	−0.0020 (12)	−0.0082 (13)
C5	0.0477 (13)	0.0327 (12)	0.0513 (15)	−0.0047 (11)	−0.0106 (13)	0.0033 (12)
C6	0.0437 (13)	0.0353 (12)	0.0515 (15)	0.0042 (11)	−0.0110 (12)	−0.0090 (12)
C7	0.083 (2)	0.0434 (16)	0.0727 (19)	0.0206 (15)	−0.0153 (16)	−0.0123 (15)
C8	0.0709 (19)	0.0503 (16)	0.0683 (17)	−0.0045 (15)	0.0121 (15)	−0.0125 (15)
C9	0.082 (2)	0.0544 (19)	0.074 (2)	−0.0040 (18)	−0.0028 (16)	0.0144 (16)

### Geometric parameters (Å, º)

**Table:**

O1—N1	1.221 (3)	C5—C6	1.378 (3)
O2—N2	1.195 (3)	C6—C7	1.518 (4)
O11—N1	1.212 (4)	C3—H3	0.9300
O22—N2	1.216 (4)	C7—H7A	0.9600
N1—C1	1.477 (3)	C7—H7B	0.9600
N2—C5	1.478 (3)	C7—H7C	0.9600
C1—C2	1.399 (4)	C8—H8A	0.9600
C1—C6	1.383 (3)	C8—H8B	0.9600
C2—C3	1.378 (4)	C8—H8C	0.9600
C2—C9	1.507 (4)	C9—H9A	0.9600
C3—C4	1.385 (4)	C9—H9B	0.9600
C4—C5	1.400 (4)	C9—H9C	0.9600
C4—C8	1.505 (4)		
			
O1—N1—O11	124.4 (2)	C5—C6—C7	122.1 (2)
O1—N1—C1	117.7 (2)	C2—C3—H3	118.00
O11—N1—C1	118.0 (2)	C4—C3—H3	118.00
O2—N2—O22	122.9 (3)	C6—C7—H7A	109.00
O2—N2—C5	119.1 (2)	C6—C7—H7B	109.00
O22—N2—C5	118.1 (2)	C6—C7—H7C	109.00
N1—C1—C2	118.0 (2)	H7A—C7—H7B	109.00
N1—C1—C6	117.6 (2)	H7A—C7—H7C	109.00
C2—C1—C6	124.5 (2)	H7B—C7—H7C	109.00
C1—C2—C3	116.0 (2)	C4—C8—H8A	109.00
C1—C2—C9	123.8 (2)	C4—C8—H8B	109.00
C3—C2—C9	120.1 (2)	C4—C8—H8C	109.00
C2—C3—C4	123.7 (2)	H8A—C8—H8B	109.00
C3—C4—C5	116.2 (2)	H8A—C8—H8C	109.00
C3—C4—C8	120.8 (2)	H8B—C8—H8C	109.00
C5—C4—C8	123.0 (2)	C2—C9—H9A	110.00
N2—C5—C4	117.3 (2)	C2—C9—H9B	109.00
N2—C5—C6	118.5 (2)	C2—C9—H9C	109.00
C4—C5—C6	124.2 (2)	H9A—C9—H9B	109.00
C1—C6—C5	115.4 (2)	H9A—C9—H9C	109.00
C1—C6—C7	122.4 (2)	H9B—C9—H9C	109.00
			
O1—N1—C1—C2	124.4 (3)	C6—C1—C2—C3	1.0 (4)
O11—N1—C1—C2	−55.6 (3)	N1—C1—C6—C5	178.6 (2)
O1—N1—C1—C6	−53.5 (3)	C1—C2—C3—C4	−2.2 (4)
O11—N1—C1—C6	126.6 (3)	C9—C2—C3—C4	178.9 (2)
O2—N2—C5—C6	−116.9 (3)	C2—C3—C4—C5	1.3 (4)
O2—N2—C5—C4	63.2 (3)	C2—C3—C4—C8	−179.9 (2)
O22—N2—C5—C4	−117.2 (3)	C3—C4—C5—C6	0.9 (4)
O22—N2—C5—C6	62.7 (3)	C8—C4—C5—N2	2.0 (4)
N1—C1—C2—C3	−176.7 (2)	C8—C4—C5—C6	−177.9 (2)
N1—C1—C6—C7	−4.4 (3)	C3—C4—C5—N2	−179.2 (2)
C2—C1—C6—C5	1.0 (3)	N2—C5—C6—C1	178.2 (2)
C2—C1—C6—C7	178.0 (2)	N2—C5—C6—C7	1.2 (4)
C6—C1—C2—C9	179.9 (2)	C4—C5—C6—C1	−1.9 (4)
N1—C1—C2—C9	2.2 (4)	C4—C5—C6—C7	−179.0 (2)

### Hydrogen-bond geometry (Å, º)

**Table:**

*D*—H···*A*	*D*—H	H···*A*	*D*···*A*	*D*—H···*A*
C7—H7*C*···O1^i^	0.96	2.60	3.232 (4)	124

Symmetry code: (i) *x*−1/2, −*y*+1/2, −*z*+1.
