# Supplementary figures and images for: Crystal structure of 1,3,5-trimethyl-2,4-di­nitro­benzene
Source: Acta Crystallogr E Crystallogr Commun. 2015 Aug 22;71(Pt 9):o670–1. doi: 10.1107/S2056989015014243 (PMC4555366; doi:10.1107/S2056989015014243)

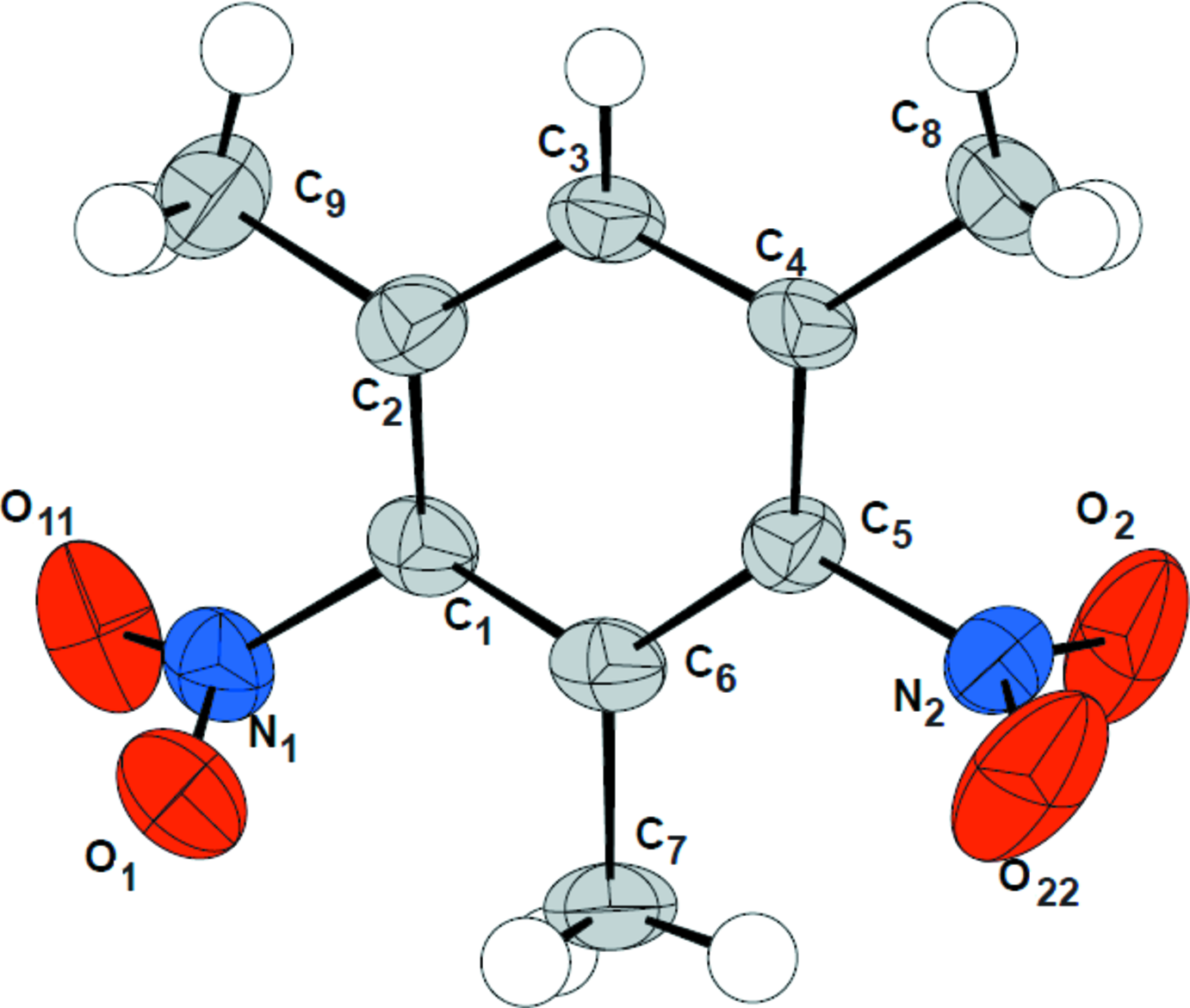

Supplement: Supplementary file 4 [file e-71-0o670-fig1.tif]

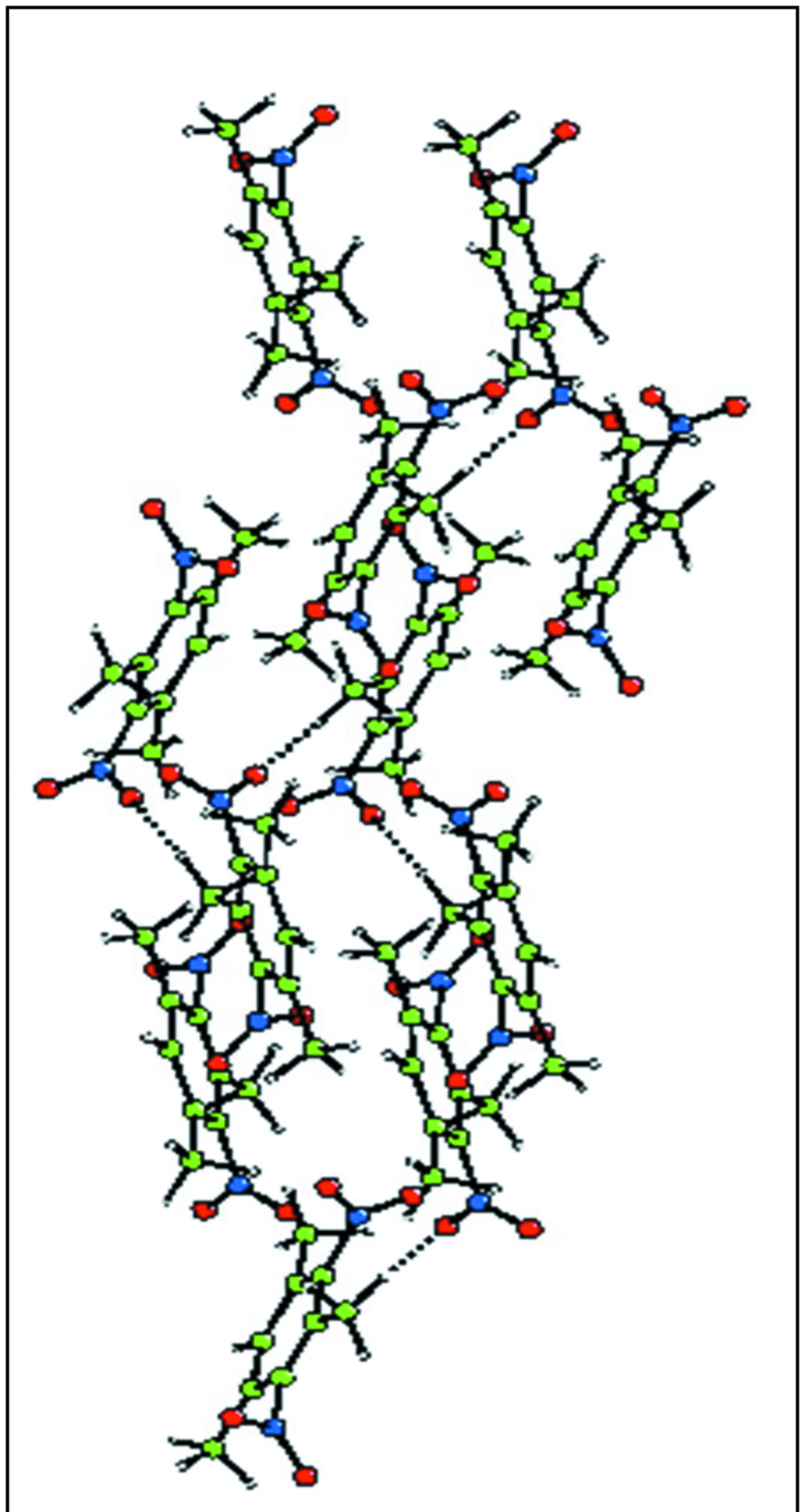

Supplement: Supplementary file 5 [file e-71-0o670-fig2.tif]

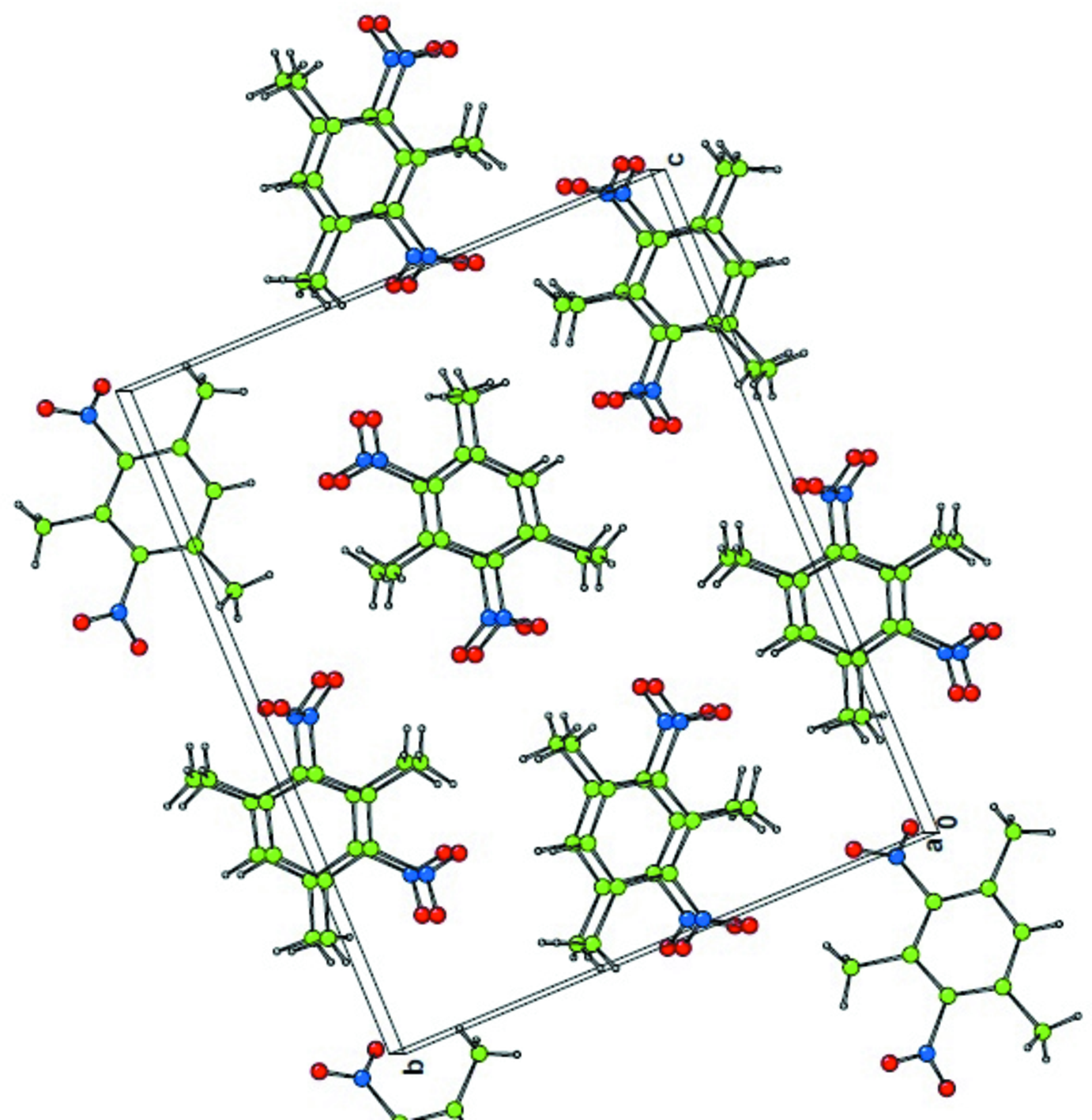

Supplement: Supplementary file 6 [file e-71-0o670-fig3.tif]
